# Relationship between Physical Fitness and Cyberbullying Patterns (Cybervictimization and Cyberperpetration) in Spanish Adolescents

**DOI:** 10.3390/bs13110952

**Published:** 2023-11-20

**Authors:** Juan de Dios Benítez-Sillero, Diego Corredor-Corredor, Luis Manuel Martínez-Aranda, Oriol Abellán-Aynés, Iago Portela-Pino, Javier Raya-González

**Affiliations:** 1 Department of Specifics Didactics, Faculty of Education Sciences and Psychology, University of Córdoba, 14071 Cordoba, Spain; eo1besij@uco.es; 2 Laboratory for Studies on Coexistence and Prevention of Violence (LAECOVI), 14071 Cordoba, Spain; 3 Counseling of Education, Junta de Andalucía, 14071 Cordoba, Spain; dcorcor015@g.educaand.es; 4 Physical and Sports Performance Research Centre, Faculty of Sports Sciences, Pablo de Olavide University, 41013 Seville, Spain; 5 SEJ-680: Science-Based Training (SBT) Research Group, Pablo de Olavide University, 41013 Seville, Spain; 6 Faculty of Sport, Catholic University of Murcia, 30107 Murcia, Spain; oabellan@ucam.edu; 7 Faculty of Health Sciences, Universidad Isabel I, 09003 Burgos, Spain; iago.portela@ui1.es; 8 Faculty of Sport Sciences, University of Extremadura, 10003 Cáceres, Spain; jrayag@unex.es

**Keywords:** cardiorespiratory, cyberbullying, strength

## Abstract

(1) Background: Cyberbullying is a growing problem among adolescents, and deeper knowledge of this phenomenon could facilitate the implementation of adequate prevention and intervention strategies. Therefore, the aim of this study was to analyze the relationships between victimization and aggression patterns in cyberbullying and physical fitness levels in Spanish adolescents. (2) Methods: A total of 741 adolescents aged 12 to 19 years from two high schools in Andalusia, Spain (mean = 14.52 ± 1.96 years; 50.9% girls, 49.1% boys) participated in the study. The participants underwent the EUROFIT battery test and completed the Spanish version of the European Cyberbullying Intervention Project Questionnaire (ECIPQ) scale. T-test, bivariate correlations and a linear regression analysis were used for statistical analysis. (3) Results: The study results indicated positive relationships between cyberbullying patterns, especially cybervictimization, and age, as well as several physical fitness measures, including BMI, sit-ups, sit-and-reach, and handgrip tests. On the other hand, cyberperpetration was positively related only to age and the specific grip strength test. Further statistical analysis revealed that cybervictimization was primarily influenced by age, while cyberperpetration was mainly influenced by age and performance in a functional test (sit-and-reach). (4) Conclusions: Cyberbullying roles, both as victims and perpetrators, may not be strongly influenced by isolated physical fitness factors. Thus, in order to reach a more comprehensive understanding and better explanations of individual involvement in cyberbullying behavior, future studies should analyze psychological and social factors along with the variables considered in this study.

## 1. Introduction

Bullying is a dynamic and complex phenomenon characterized by intentional interpersonal aggression that is repetitive and sustained over time, primarily occurring among children or adolescents [1]. These behaviors often manifest within the school environment and involve a power imbalance and dominance dynamic between the perpetrator and the victim [2]. However, it is worth noting that individual personalities also play a significant role in these interactions [3]. Notably, some previous studies have indicated that victims of traditional bullying tend to have higher rates of overweight and a higher body mass index (BMI) compared to their non-victimized peers [4]. Likewise, perpetrators of traditional bullying engage in physical behaviors such as hitting, pushing, and insulting, often within close physical proximity to the victim [5]. These findings suggest that physical differences, especially those related to aspects of physical fitness, may influence the roles of bullying. Addressing bullying situations promptly is imperative, as they can lead to reiterative and recurrent issues over time, such as increased stress and anxiety, decreased self-esteem and self-confidence, deterioration of self-concept, and so on, resulting in physical, psychological, and social health problems for the victims [6]. Therefore, it is essential to gain a comprehensive understanding of the characteristics of both the victim and the perpetrator to establish effective preventive and corrective measures [7]. However, it is an undeniable fact that the lifestyles of young people have evolved in recent years, with a significant increase in mobile phone and internet usage [8]. This transformation has had a profound impact on interpersonal relationships [9], even leading to the emergence of negative behaviors [10], among which cyberbullying stands out [11]. Consequently, expanding our knowledge of this modern form of harassment is crucial to developing tailored prevention strategies for different contexts.

Cyberbullying represents a contemporary form of harassment that is perpetrated either individually or by groups through electronic devices, primarily targeting individuals who may find it challenging to defend themselves [12]. Various motivations have been identified for engaging in cyberbullying, including factors like revenge, envy, prejudice, intolerance, shame, pride, guilt, and anger [13]. Cyberbullying differs from traditional bullying in several key characteristics, such as the potential for anonymity on the part of the perpetrator, a wide audience reached with rapid execution and dissemination, and the ability to occur without direct confrontation with the victim, anytime and anywhere [14]. Nevertheless, both forms of harassment share common features, notably intentionality, repetition, and an imbalance of power [15]. When considering preventive measures for cyberbullying, it is important to acknowledge that this form of harassment enables aggressions through various means, with text messages, phone calls, or social networks and websites being the most common platforms [16]. Given the recent surge in cyberbullying among young populations [17], as well as its frequent association with traditional bullying [18], cyberbullying has become a significant social concern. Therefore, it is imperative to conduct research and explore effective strategies to combat this evolving phenomenon, to better safeguard the well-being of individuals, especially those who may be vulnerable to its effects.

One noteworthy strategy that has emerged for combating both traditional bullying and cyberbullying is regular physical activity [19]. Previous research has demonstrated that engaging in consistent physical exercise can yield multiple psychological benefits, including increased self-control, heightened self-esteem, and enhanced empathy, all of which are associated with a decrease in aggressive behavior [19,20]. For example, Merrill et al. [19] observed a lower prevalence of cyberbullying among students who were physically active and engaged in at least 60 min of physical exercise on at least five days per week, as compared to those who engaged in physical activity less frequently. Similarly, Sibold et al. [21] revealed that higher levels of weekly physical activity were linked to reduced levels of victimization among students aged 14–18 years. Furthermore, some studies have explored the connections between physical fitness levels and victimization and aggression in the context of bullying. In this regard, Hormonal-Aguayo et al. [22] established a significant relationship between cardiorespiratory fitness levels and lower victimization rates in traditional bullying. Likewise, García-Hermoso et al. [23] found that non-victimized adolescents involved in traditional bullying exhibited higher levels of cardiorespiratory fitness compared to their victimized counterparts. Nevertheless, it is important to note that there is still a need for additional research to comprehensively examine the relationships between physical fitness levels and cyberbullying in both victim and perpetrator roles. This could provide valuable insights into how physical activity can be leveraged to address the complex dynamics of cyberbullying and contribute to the development of effective preventive measures.

Despite the valuable information on the relationship between physical fitness levels and bullying, and the significant differences between traditional bullying and cyberbullying, further studies on this topic are necessary. Therefore, the aim of this study was to analyze the relationships between victimization and aggression patterns in cyberbullying and physical fitness levels in Spanish adolescents. Based on prior studies [22,23], we hypothesized that there is a relationship between cyberbullying and physical fitness levels in both roles, although it is possible that this relationship may not be highly determinant in cyberperpetration due to the specific characteristics of cyberbullying.

## 2. Materials and Methods

### 2.1. Study Design

A comprehensive descriptive and correlational study was carried out to thoroughly analyze the relationships between the physical fitness levels of adolescents and their experiences with cybervictimization and cyberperpetration. Participants were engaged in the EUROFIT battery assessment over the course of three separate days during the school period. Additionally, the Spanish version of the European Cyberbullying Intervention Project Questionnaire (ECIPQ) scale was administered using both classroom computers and mobile devices, with each assessment session lasting between 20 to 30 min. This study was conducted as a part of a project funded and authorized by the Directorate General for Innovation and Teacher Training of the Andalusian Department of Education as an educational research project.

### 2.2. Participants

A total of 741 adolescents, ranging in age from 12 to 19 years (mean age = 14.84; standard deviation = 1.68 years) actively participated in this study. These adolescents were selected through a convenience sampling method and were drawn from two high schools situated in northern Andalusia, Spain, with a gender distribution of 377 female students (50.9%) and 364 male students (49.1%). All subjects gave their informed consent for inclusion before they participated in the study. Participants were provided with a clear understanding of its objectives, emphasizing that the participation would be completely anonymous, confidential, and entirely voluntary. The research project received the necessary permissions from the school authorities, and the families of the participants formally consented to their involvement. The study was conducted in accordance with the Declaration of Helsinki, and the protocol was approved by the Ethics Committee of the Universidad de Córdoba, Spain, (PIV-034/18) at 11 December 2019.

### 2.3. Procedures

#### 2.3.1. Anthropometric Measures

Anthropometric data were gathered on the initial day of assessment, with adolescents being measured in a barefoot and lightly clothed state. To determine weight in kilograms (kg), a Tanita BF 350 scale was utilized, boasting an impressive accuracy level of 0.1 kg. Furthermore, height measurements were conducted using a SECA stadiometer, known for its precision, with an accuracy of 0.1 cm. In addition to these measurements, the body mass index (BMI) was calculated for each participant. The BMI was derived using the formula BMI = weight (kg)/height (m^2^). BMI was classified according to the following scale: normal weight (18.5–24.9 kg/m^2^), overweight (25.0–29.9 kg/m^2^), obesity class I (30.0–34.9 kg/m^2^), and obesity class II (35.0–39.9 kg/m^2^).

#### 2.3.2. Physical Fitness Tests

The EUROFIT battery, a well-established and validated fitness assessment tool, was administered over the course of three days as part of this study [24]. This battery of fitness tests has been widely employed to evaluate the physical fitness levels of adolescents and is considered a standard in the field. In addition, it can be used as a reliable battery of tests in order to assess physical fitness in research and practice [25]. The tests included in the EUROFIT battery are not only recognized but are also familiar to the students as they were routinely incorporated into their educational assessment toolkit [26]. This battery provides a comprehensive evaluation of various aspects of physical fitness, helping researchers gain valuable insights into the adolescents’ overall fitness levels and capabilities. The description of the tests is as follows:

**(a) A 30 s sit-up test:** This test is employed to evaluate muscular endurance among participants. To perform this test, individuals lie face up on a mat, positioning their hands behind the back of their neck. Their legs are flexed at a 90-degree angle with their feet supported. The aim is for participants to complete as many sit-ups as possible within a 30-second timeframe while ensuring that they touch their knees with their elbows during each sit-up repetition. A Casio HS-80TW stopwatch was used to accurately time test duration, and the number of correctly executed sit-up movements was recorded. This test is valuable in gauging the endurance of the participants’ abdominal and core muscles, providing insights into their physical fitness levels and their capacity for sustaining repeated muscle efforts.

**(b) Sit-and-reach flexibility test:** This test is designed to assess the range of motion of the hip joint. Participants are instructed to reach as far as they can, measured in cm, using their extended hands. They move their fingertips along a ruler positioned on the surface of a standardized box equipped with an adhesive tape measure. To perform this test, participants are seated on a mat with their legs fully extended, and the soles of their feet are placed in a standardized position on the box. The procedure involves flexing the trunk forward while keeping the legs extended. It is important to note that, as per the protocol, a fixed measure of 15 cm is placed in the position where the participants’ feet rest. This standardized setup ensures consistency in the test conditions and allows for an accurate assessment of hip joint flexibility. By measuring how far participants can reach, this test provides valuable information about their hip joint mobility and flexibility.

**(c) Horizontal jump:** This test is aimed at assessing the ballistic muscular strength of the lower limbs. Participants begin in a stationary position, directly behind a designated starting line, with their feet positioned shoulder-width apart and parallel to each other. The objective is for them to execute a powerful jump forward, maximizing the distance covered, all while maintaining balance upon landing. The distance of their jump was measured in cm using an adhesive tape placed on the ground. Each participant was given two opportunities to perform the jump, and the better of the two attempts, which resulted in a longer distance, was chosen and recorded. This test is an effective means of gauging the explosive strength and power in the lower body, providing valuable insights into the participants’ muscular capabilities.

**(d) A 20 m Multistage Shuttle Run Test (SRT):** This test indirectly assesses the maximum aerobic capacity of the participants. It involves a maximal incremental run conducted over a 20 m distance, utilizing an out-and-back course. This test is facilitated by specialized software in MP3 format, as originally designed by Léger et al. [27]. The test initiates at a moderate pace, and participants are tasked with adjusting their direction in response to sound signals that become increasingly rapid. The intention is to gradually accelerate the pace throughout the test. Participants continue with this pattern until they are no longer able to sustain the prescribed pace. To determine the performance, the time taken to complete the test was measured, recorded in min and s. This precise measurement ensures an accurate evaluation of the participants’ maximum aerobic capacity, shedding light on their cardiovascular fitness and endurance levels.

**(e) Handgrip strength:** The purpose of this test is to evaluate the strength of the upper limbs. In this assessment, participants make use of a TAKEY TKK 5110 dynamometer equipped with an adjustable handle. Their task is to apply gradual and continuous pressure to the dynamometer for a duration of 2 s, aiming to generate the maximum strength they can apply. The strength exhibited is measured in kg with an accuracy level of 0.1 kg. Each participant was provided with two opportunities to perform this task with each hand, and the results from these attempts were recorded. The best results, showcasing the highest level of upper limb strength achieved, were then selected for analysis. This test provides valuable insights into the participants’ upper body strength capabilities and is an essential component of assessing their overall physical fitness.

To minimize any potential interference between the tests and to maintain the integrity of the measurements, a carefully planned sequence of assessments was conducted on different days. This approach was designed to ensure that participants had ample time to recover between each evaluation. The testing schedule was as follows: On day one, the sit-and-reach flexibility test and sit-up test were conducted. On day two, a 20 m shuttle run (Course Navette) was performed. On day three, handgrip strength and horizontal jump tests were performed. This thoughtfully planned sequencing of tests on different days adheres to best practices in fitness assessment, as it allows for consistent and reliable measurements while safeguarding against any potential performance fluctuations due to fatigue or exertion from previous tests [28].

#### 2.3.3. European Cyberbullying Intervention Project Questionnaire (ECIPQ)

The Spanish version [29] of the European Cyberbullying Intervention Project Questionnaire (ECIPQ) [30] was implemented for cyberbullying assessment. This questionnaire comprises 22 items (11 based on cybervictimization and 11 based on cyberperpetration) which were evaluated using a Likert-type scale with five response options (from 0 to 4, with 0 = never, 1 = once or twice, 2 = once or twice a month, 3 = about once a week, and 4 = more than once a week). For both dimensions, the items refer to actions such as swearing, excluding or spreading rumors, impersonating, etc., all of them in electronic media and referring to a time interval of the last two months. Some of the items are “Someone has called me names or insulted me on the Internet” and “I have posted compromising videos or photos of someone on the Internet”. The internal consistency (α) values of the test were 0.86 for cybervictimization and 0.87 for cyberperpetration.

### 2.4. Statistical Analysis

Descriptive data were presented as mean ± standard deviations (SD). To compare the variables based on gender, an independent-samples *t*-test was performed. Additionally, we calculated effect sizes according to Cohen’s guidelines [31], categorized as follows: <0.2, trivial; 0.20 to 0.49, small; 0.50 to 0.80, moderate, and >0.80, large. Subsequently, bivariate correlations were performed using Spearman’s test to explore the relationships between physical fitness levels and cybervictimization as well as cyberperpetration. Furthermore, a linear regression analysis was carried out, with cybervictimization and cyberperpetration serving as the dependent variables, and the results of the physical fitness tests as the independent variables. Prior to the regression analysis, we conducted a Variance Inflation Factors (VIF) test to detect multicollinearity, interpreted as follows: <5, low correlation between variables; 5 to 10, moderate correlation between variables; and >10, moderate correlation between variables, considered as not tolerable correlation of model predictors [32]. The statistical significance was established at *p* < 0.05, and the data analysis was carried out using the Statistical Package for Social Sciences (SPSS 25.0; SPSS Inc., Chicago, IL, USA).

## 3. Results

Between-gender differences are presented in Table 1. Males exhibited better performance in the sit-up test, horizontal jump test, 20 m Multistage Shuttle Run Test, and handgrip strength test, while females demonstrated better performance in the sit-and-reach flexibility test.

In Table 2 is shown the linear correlations between physical fitness tests and cybervictimization and cyberperpetration. Cybervictimization showed positive relationships with age, BMI, sit-up, sit-and-reach, and handgrip. For cyberperpetration, positive relationships were found with age and handgrip.

The results derived from the linear regression analysis are presented in Table 3. The VIF analysis revealed values <5 for all variables (i.e., between 1.143 and 3.191), indicating a low correlation between variables. Cybervictimization was influenced by age and the sit-up test. The cybervictimization model as a dependent variable was found to be very significant (*p* = 0.005; F = 2.760; R^2^ = 0.029). Furthermore, cyberperpetration was influenced by age and the sit-and-reach test, finding a significant relationship. In this case, within the cyberperpetration model, the dependent variable was clearly significant (*p* = 0.009; F = 2.559; R^2^ = 0.027).

## 4. Discussion

This study aimed to analyze the relationships between victimization and aggression patterns in cyberbullying and physical fitness level in Spanish adolescents. The main novelty of this study was that analyze the aforementioned relationships considering cyberbullying instead bullying. The obtained results revealed positive relationships for cybervictimization with age, BMI, sit-up, sit-and-reach, and handgrip, while cyberperpetration was related to age and handgrip. Additionally, the linear regression analysis showed that cybervictimization was influenced by age and sit-up, while cyberperpetration was influenced by age and sit-and-reach.

Traditionally, bullying victims have been associated with deficiencies in certain psychological variables, such as self-esteem, self-concept or poor social relationships [33]. Likewise, the evidence on the relationship between being a victim of traditional bullying with being overweight and have a higher BMI is quite consolidated [4]. However, in this study, once the linear regression models were considered, there were no relationships between BMI with cybervictimization. This finding does not support the results obtained in some studies, such as the study conducted by Lessard and Puhl [34]. However, other studies have analyzed the association between BMI and cybervictimization, and the relationship is much weaker than in the case of traditional bullying, with no significant effect of BMI on the probability of being bullied online [35]. We hypothesize that in traditional bullying, face to face, the main resource is to attack physical appearance, whereas in cyberbullying, when behind the screen, the resources to victimize another subject seem to be more focused on other types of less-obvious variables, and related to behavioral and social factors.

Meanwhile, in recent years, a relationship has been observed between the possibility of being bullied and the level of physical fitness [2]. Specifically, García-Hermoso et al. [23] and Hormazábal-Aguayo et al. [22] observed a lower incidence of victimization in boys and girls with higher values of cardiorespiratory fitness in traditional bullying. However, the differences in cyberbullying make it pertinent to expect different results. In this study, cybervictimization was found to be positively related to age, BMI, sit-up, sit-and-reach, and handgrip. Notably, age and sit-up were the only variables that significantly influenced cybervictimization in the regression model. This could be related not only to the influence of age itself (as a sensitive stage within the individual’s developmental process), but also to the fact that adolescents acquire greater digital skills and access to these devices as they grow older, exposing them to higher risks [8]. Conversely, traditional bullying is more related to victimization behaviors such as hitting, pushing, insulting, etc., which occur during the closest physical interaction [5]. In these behaviors, physical differences can be evident in relation to body weight, with the components of physical condition related to strength being those that seem to have a greater relationship with these behaviors. Therefore, it seems recommendable in general terms to implement control strategies and promote responsible use of technologies to reduce the incidence of cybervictimization among school adolescents.

As occurs with victimization in traditional bullying, overweight subjects are more involved in traditional perpetration [36], these relationships also having associations with mental health problems such as social phobia, self-esteem, or depression among others [37]. However, in our study no relationships were found, either at the correlational level or in the linear regression model, between cyberperpetration and BMI. In addition, it is necessary to highlight that our study did not categorize adolescents who were overweight or obese compared to those who did not suffer from it. This was already done in the aforementioned studies [36,37], which could explain these differences. Regarding the role of cyberperpetration, prior studies have suggested that bullies tend to exhibit higher sensitivity to reward, larger communicational and relational skills, and lower emotional sensitivity [3]. In relation to physical fitness attributes, Benítez-Sillero et al. [2] concluded that muscular strength, especially in boys, could be an important predictor of aggression in bullying. However, in the context of cyberbullying, our study found a positive relationship between cyberperpetration and age and handgrip, although in the linear regression model, only age and the sit-and-reach test showed significant influence on cyberperpetration. These findings suggest a fundamental shift in the dynamics of aggression in the digital realm compared to traditional physical bullying. In the context of cyberbullying, physical prowess loses its relevance, as perpetrators can hide behind the anonymity of computer screens, without the need to demonstrate physical superiority to perpetrate cyberbullying [8]. Unlike the tangible manifestations of aggression in traditional bullying, where physical disparities like strength or size often dictate power dynamics, and manifest physical differences occur such as hitting or pushing [5], the digital world creates an environment where these factors become less significant. Indeed, cyberaggressors wield influence through different means, such as verbal attacks, spreading malicious content, or psychological manipulation, making their actions no less harmful but distinct in nature. The absence of direct physical confrontation in cyberbullying also means that traditional deterrents, like physical intervention, are largely ineffective. Consequently, combating cyberbullying requires a different set of strategies, including education, online safety measures, and fostering a culture of digital empathy and respect. Understanding these nuances is crucial in addressing the evolving landscape of aggression in the digital age.

On the other hand, it is striking that higher levels of cyberperpetration were related to worse levels of flexibility through the sit-and-reach test. This could be related to the greater skills in digital competence of those who present higher levels of cyberaggression [8], which could be associated with a greater time dedicated to activities related to the new sedentary technologies [38]. The greater dedication to activities related to new technologies means that the time spent sitting is longer [39], which could be related to lower levels of flexibility of the posterior muscle chain that is evaluated through the sit-and-reach test [26]. Some studies have found worse levels of flexibility, as measured by this test, in adolescents who spend more time outside the classroom using new technologies [40].

This study presents some limitations that need to be highlighted. Firstly, the time each adolescent spent on technology was not recorded, which could potentially influence the results obtained. Additionally, the study only considered physical variables, neglecting the potential influence of psychological variables that could provide valuable insights into the complex nature of cyberbullying behaviors. Moreover, the study missed considering other variables related to gender or academic performance, which could offer valuable information to better understand the characteristics of the different roles in cyberbullying.

## 5. Conclusions

The results of the study indicated positive relationships between cybervictimization and age, as well as several physical fitness measures, including BMI, sit-ups, sit-and-reach, and handgrip. On the other hand, cyberperpetration was positively related to age and handgrip. The linear regression analysis further revealed that cybervictimization was primarily influenced by age and sit-up, while cyberperpetration was influenced by age and performance in the sit-and-reach test. These findings suggest that the roles of cyberbullying, both as victims and perpetrators, may not be strongly influenced by physical fitness factors in isolation. Instead, other variables, such as psychological factors and social dynamics, could play a more significant role in determining an individual’s involvement in cyberbullying. Therefore, in order to obtain a more comprehensive understanding of cyberbullying, future studies should consider a broader range of variables, including both physical and psychological aspects. Such studies will help in developing more effective preventive strategies and interventions to address cyberbullying in schools and online environments.

## Figures and Tables

**Table 1:** Descriptive data and between gender comparisons.

Variables	Males (n = 364)	Females (n = 377)	Comparison	
	M (SD)	M (SD)	*p*	d
BMI (kg·m^−2^)	21.99 (4.48)	22.00 (4.08)	0.496	0.002
Sit-up (n)	25.50 (6.62)	20.52 (5.56)	0.001	0.752
Sit-and-reach (cm)	13.51 (7.90)	18.20 (8.86)	0.001	0.594
Horizontal jump (cm)	183.31 (37.10)	141.00 (27.10)	0.001	1.140
Shuttle Run Test (periods)	5.76 (2.72)	3.66 (1.60)	0.001	0.772
Handgrip (kg)	55.66 (17.10)	41.52 (9.56)	0.001	0.823
Cybervictimization	0.15 (0.36)	0.16 (0.28)	0.323	0.028
Cyberperpetration	0.10 (0.26)	0.08 (0.28)	0.179	0.077

Abbreviations: n = number of participants; M = mean; SD; standard deviation. Significance level at *p* < 0.05.

**Table 2:** Linear correlations between fitness tests and victimization and aggression in cyberbullying.

Participants	Cybervictimization	Cyberperpetration
Age	0.245 ***	0.210 ***
BMI	0.076 *	0.044
Sit-up	0.103 **	0.057
Sit-and-reach	0.082 *	0.009
Horizontal jump	0.019	0.059
Shuttle Run Test	0.016	0.029
Handgrip	0.106 **	0.107 **

Abbreviations: BMI = body mass index. * *p* < 0.05; ** *p* < 0.01; *** *p* < 0.001.

**Table 3:** Linear regression models for cybervictimization and cyberperpetration.

Variables	Cybervictimization		Cyberperpetration	
	β	t	β	t
Gender	0.010	0.332	0.019	0.741
Age	0.023	2.738 **	0.016	2.375 *
BMI	0.000	0.126	−0.001	−0.529
Sit-up	0.005	2.168 *	0.003	1.723
Sit-and-reach	0.002	1.262	−0.003	−2.152 *
Horizontal jump	0.000	−0.245	0.000	−0.308
Shuttle Run Test	−0.011	−1.734	−0.002	−0.429
Handgrip	0.000	0.128	0.001	0.868

* *p* < 0.05; ** *p* < 0.01.

## Data Availability

Data can be requested from the corresponding author.
